# The influence of size, clearance, cartilage properties, thickness and hemiarthroplasty on the contact mechanics of the hip joint with biphasic layers^☆^

**DOI:** 10.1016/j.jbiomech.2013.04.009

**Published:** 2013-06-21

**Authors:** Junyan Li, Todd D. Stewart, Zhongmin Jin, Ruth K. Wilcox, John Fisher

**Affiliations:** a Institute of Medical and Biological Engineering, School of Mechanical Engineering, University of Leeds, UK; b School of Mechanical Engineering, Xi'an Jiaotong University, People's Republic of China

**Keywords:** Contact mechanics, Articular cartilage, Biphasic, Hip, Finite element

## Abstract

Computational models of the natural hip joint are needed to examine and optimise tissue sparing interventions where the natural cartilage remains part of the bearing surfaces. Although the importance of interstitial fluid pressurisation in the performance of cartilage has long been recognized, few studies have investigated the time dependent interstitial fluid pressurisation in a three dimensional natural hip joint model. The primary aim of this study was to develop a finite element model of the natural hip incorporating the biphasic cartilage layers that was capable of simulating the joint response over a prolonged physiological loading period. An initial set of sensitivity studies were also undertaken to investigate the influence of hip size, clearance, cartilage properties, thickness and hemiarthroplasty on the contact mechanics of the joint. The contact stress, contact area, fluid pressure and fluid support ratio were calculated and cross-compared between models with different parameters to evaluate their influence. It was found that the model predictions for the period soon after loading were sensitive to the hip size, clearance, cartilage aggregate modulus, thickness and hemiarthroplasty, while the time dependent behaviour over 3000 s was influenced by the hip clearance and cartilage aggregate modulus, permeability, thickness and hemiarthroplasty. The modelling methods developed in this study provide a basic platform for biphasic simulation of the whole hip joint onto which more sophisticated material models or other input parameters could be added in the future.

## 1 Introduction

Articular cartilage comprises two principal phases: a solid phase which includes collagen fibrils and proteoglycan macromolecules, and a water-like fluid phase. The importance of interstitial fluid pressurisation on the behaviour of cartilage has been known for decades (Mow et al., 1980, 1984, Ateshian et al., 1994). It has been proven that osteoarthritis (OA) is related to not only the magnitude but also the duration of contact stress (Hadley et al., 1990, Maxian et al., 1995), both of which are closely linked to the mechanical behaviour of the interstitial fluid in the cartilage (Ateshian et al., 1994). In order to study the biotribology of articular joints such as the hip, and to understand the changes that occur with degeneration and potential interventions, it is therefore necessary to consider the biphasic nature of the cartilage within the joint system.

Experimental measurements of articular joint contact mechanics can provide valuable information, but they involve highly invasive techniques such as the insertion of transducers (Brown and Shaw, 1983, Hodge et al., 1989) or pressure-sensitive film (Afoke et al., 1987) into the joint. These methods may introduce measurement artefacts between articular surfaces and thus affect the results (Brand et al., 2001). Moreover, the parameters that can be measured are limited. For instance, direct measurement of fluid pressure distribution inside the cartilage of the natural hip joint is currently difficult and has only been achieved for very simple configurations (Soltz and Ateshian, 1998, Park et al., 2003).

Numerical analysis serves as an alternative approach. However, existing models assume the cartilage to be either elastic or hyperelastic (Yoshida et al., 2006, Anderson et al., 2008, Chegini et al., 2009, Anderson et al., 2010, Harris et al., 2012), which cannot account for the interstitial fluid flow in the cartilage. The loss of load support by the fluid in the cartilage is believed to be one reason for the increased coefficient of friction and higher shear stress which may lead to progressive degradation in the cartilage and onset of hip OA (Forster and Fisher, 1996, Mccann et al., (2009)). Biphasic modelling is able to account for the fluid flow in the cartilage providing more information on the contact mechanics and tribology for the natural hip joint. Several numerical studies on the investigation of the labrum have adopted biphasic soft tissues for two dimensional hip models (Ferguson et al., 2000a, 2000b, Haemer et al., 2012). Recently, Pawaskar et al. (2010) developed a three dimensional hemiarthroplasty hip model incorporating biphasic cartilage layers on the acetabulum using Abaqus (version 6.7-1, DassaultSystemes, SuresnesCedex, France) and applied the model to the simulation of daily activities for several cycles. However, for biphasic cartilage-on-cartilage contact, especially in the case of whole joints, there are difficulties in sustaining convergence of the model for prolonged periods of physiological loading using this software. As yet, the biphasic approach does not appear to have been applied to three dimensional modelling of the natural hip joint to examine the contact mechanics over a prolonged physiological period of loading.

It is widely realized that the congruence and size of the human hip joint and the material properties of the hip cartilage vary between individuals (Athanasiou et al., 1994, von Eisenhart et al., 1999, Shepherd and Seedhom, 1999, Xi et al., 2003). However, to what extent and how these parameters influence the contact mechanics of the natural hip joint are not fully understood. Besides, the influence of hemiarthroplasty (e.g. femoral head replaced with metallic prosthesis if only the femoral head cartilage breaks down (Pawaskar, 2010)) on the hip function under prolonged physiological periods of loads is unclear. Quantifying these influences can serve to better understand the hip function as well as to identify the accuracy of measurements needed for the development of future subject-specific computational models of the hip and their validation.

The primary aim of this study was therefore to develop a finite element (FE) model of the natural hip incorporating the biphasic cartilage layers that was capable of simulating the joint response over a prolonged physiological loading period. In order to investigate the role of the parameters within this model, a set of sensitivity studies were then undertaken to evaluate the influence of hip size, clearance, cartilage properties, thickness and hemiarthroplasty on the contact mechanics of the joint.

## 2 Methods

**Fig. 1:**
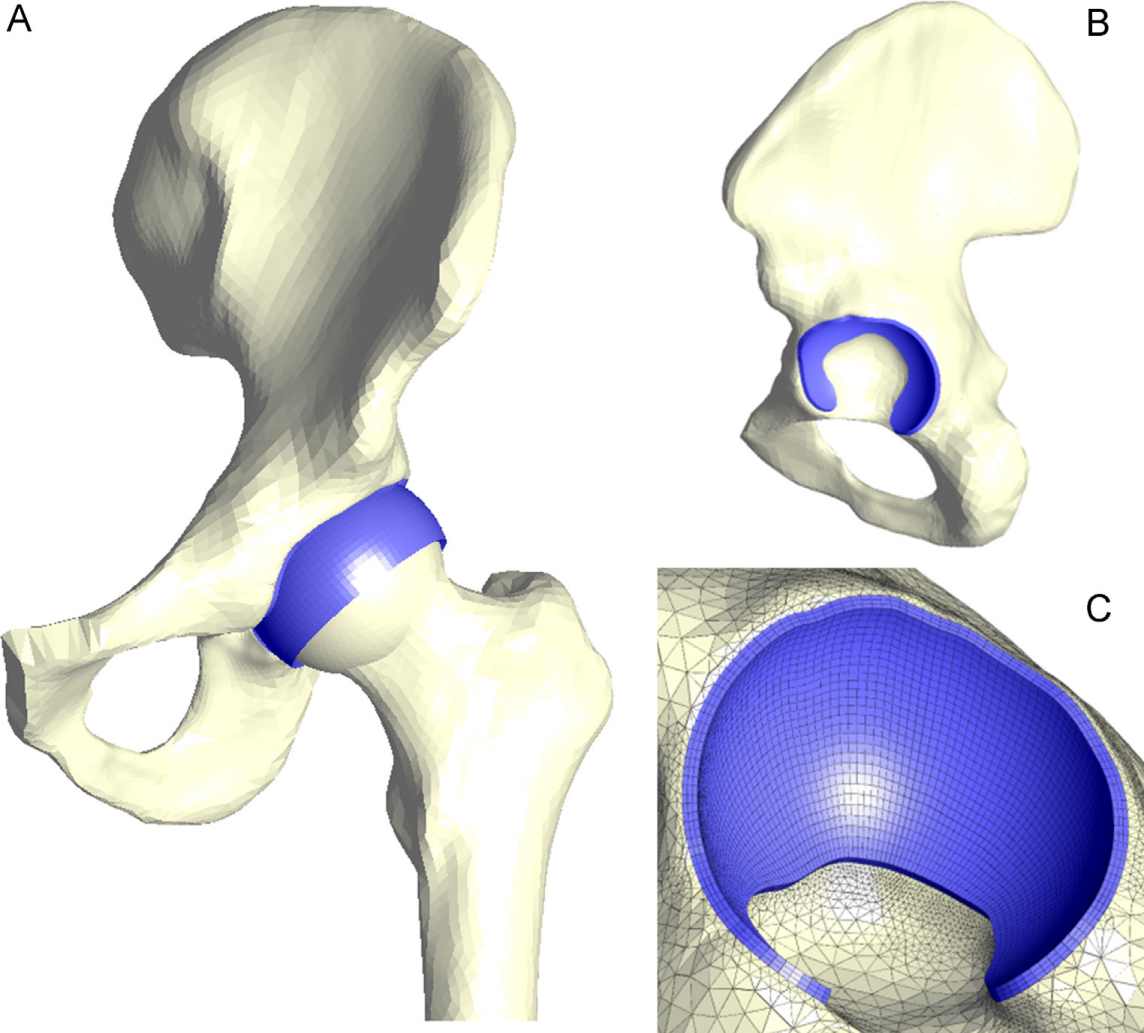
The three dimensional finite element model of the hip joint. A–The entire model, B–Lateral view of acetabulum. C–Oblique view of acetabular cartilage with hexahedral elements.

The model utilized in the study was based on a standardized solid model of the pelvis and femur from a 38 year-old healthy human male at the time of death, available from the Internet through the BEL repository (Author: Vicceconti, from: www.tecno.ior.it/VRLAB/). The acetabulum and the femoral head surfaces were carefully trimmed spherically (Hammond and Charnley, 1967, Rushfeldt et al., 1981), and a layer of cartilage with uniform thickness was created from the spherical area. The resultant model approximated the native horseshoe shaped acetabular cartilage and the femoral head cartilage coverage (Fig. 1). The geometric model and corresponding FE model were generated using I-DEAS (Version 6.1, Siemens PLM Software Inc., Plano, USA). The bone components of the femur and pelvis were meshed with around 135,000 four-noded tetrahedral elements. The femoral head and acetabular cartilage layers were made up of around 5700 and 8400 eight-noded hexahedral elements respectively. The bone was meshed based on the elements of the acetabular cartilage so that the surface of the subchondral bone shared the same nodes as the inner surface of the cartilage layer. The mesh density was evaluated to ensure that the differences in the peak contact stress, peak fluid pressure and fluid support ratio (the load supported by the fluid pressure over the total load) were less than 5% when the number of elements was doubled.

The material properties and geometric parameters associated with the cartilage were initially taken from the literature and were then sequentially varied in a parametric study (Table 1). The models with varied geometric parameters (i.e. size, clearance or cartilage thickness) were achieved by scaling the spherically trimmed femur and pelvis and subsequently recreating the cartilage layers. A hemiarthroplasty model was also generated which had identical geometric parameters to the original model, with the femoral head replaced by an impermeable sphere representing a metal prosthesis. The cartilage was modelled as a biphasic solid and the solid phase was represented as neo-Hookean, with the following strain energy (*W*) given in (Maas and Weiss, 2007).

$$W=\frac{\mu }{2}(I_{1}-3)-\mu \ln\;J+\frac{\lambda }{2}{\left(\ln\;J\right)}^{2}$$

where, *μ* and *λ* are the Lamé parameters; *J* volume ratio; *I*_1_ first strain invariant of the deviatoric Cauchy–Green tensor *C*.

**Fig. 2:**
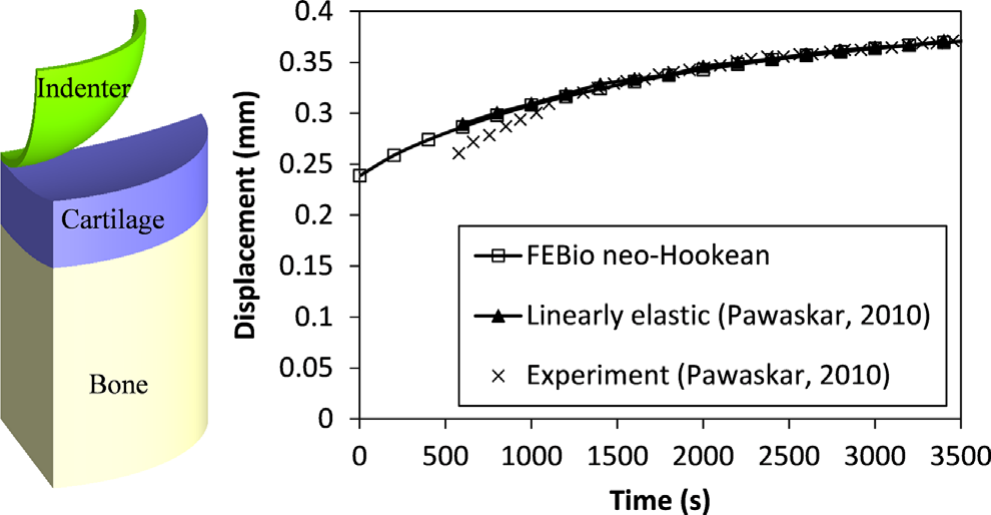
Verificaiton of the constitutive properties (left**–**model; right**–**results). Indentation model of a creep test using a quarter-symmetry model. Material properties and geometric parameters were taken from a previous study (Pawaskar, 2010). The biphasic model with neo-Hookean solid phase in FEBio behaves nearly identically to the biphasic model with linearly elastic solid phase in Abaqus (Maas and Weiss, 2007). The experimental results from Pawaskar (2010) are also shown.

The methodology and material constitutive relationship was verified on an indention model against a linearly elastic material model developed in Abaqus (Pawaskar, 2010), and both predicted similar time-dependent behaviour (Fig. 2). The Poisson's ratio of the aggregate was 0.045; this value has been shown to have little influence on the results when varied from 0 to 0.1 (Athanasiou et al., 1994).

The bone was modelled as impermeable and linearly elastic with a Young's modulus of 17000 MPa and Poisson's ratio of 0.3 (Dalstra and Huiskes, 1995). The cortical bone and trabecular bone were not modelled separately because it was found that changes in the peak contact stress and peak fluid pressure were within 5% if the Young's modulus of the whole region was reduced from that representing all cortical bone (17000 MPa) to that representing all trabecular bone (800 MPa).

Nodes at the sacroiliac and pubis symphysis joints were fixed in all degrees of freedom. The contact between articulating surfaces was assumed to be frictionless. For the models of natural joints, the contact formulation allowed fluid to flow between contacting surfaces as well as from open surfaces of the cartilage. No fluid flow was allowed through the contact-against-rigid surfaces of the acetabular cartilage in the hemiarthroplasty model. A static load of approximately 2130 N, based on the average data for one leg stance (Bergmann et al., 2001), was applied to the distal femur, which was constrained in rotational degrees of freedom. The load was ramped over 0.6 s and then held constant for 3000 s.

All analyses were conducted using the open-source non-linear FE solver FEBio (version 1.5.0; mrl.sci.utah.edu/software/febio) (Maas et al., 2012) due to its good convergence ability in the simulation of biphasic materials in contact. The models were solved on a Linux server with 8 GB of RAM and 8 Intel X5560 cores at 2.8 GHz. Contact stress, contact area, fluid pressure and fluid support ratio were recorded over the time period from 0 to 3000 s to evaluate the load transmission and tribological performance.

## 3 Results

**Fig. 3:**
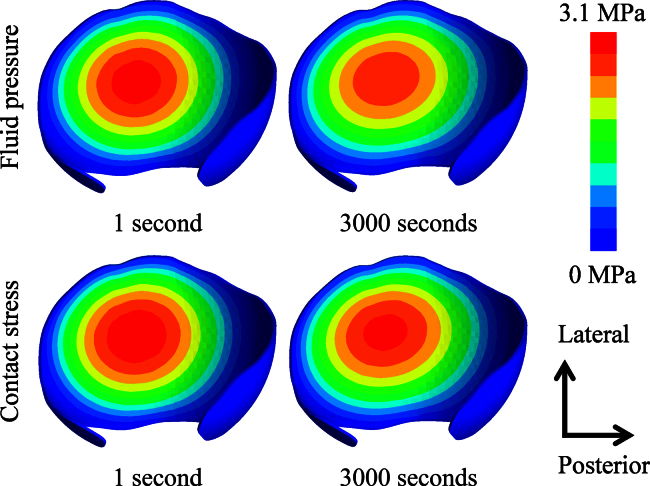
Contours of fluid pressure (MPa) and contact stress (MPa) of the acetabular cartilage for the original model at 1 s and 3000 s. On the acetabular cartilage surface, the peak contact stress is slightly higher than the peak fluid pressure. Obvious cartilage consolidation can be detected. The change in the fluid pressure is greater than that in the contact stress.

**Fig. 4:**
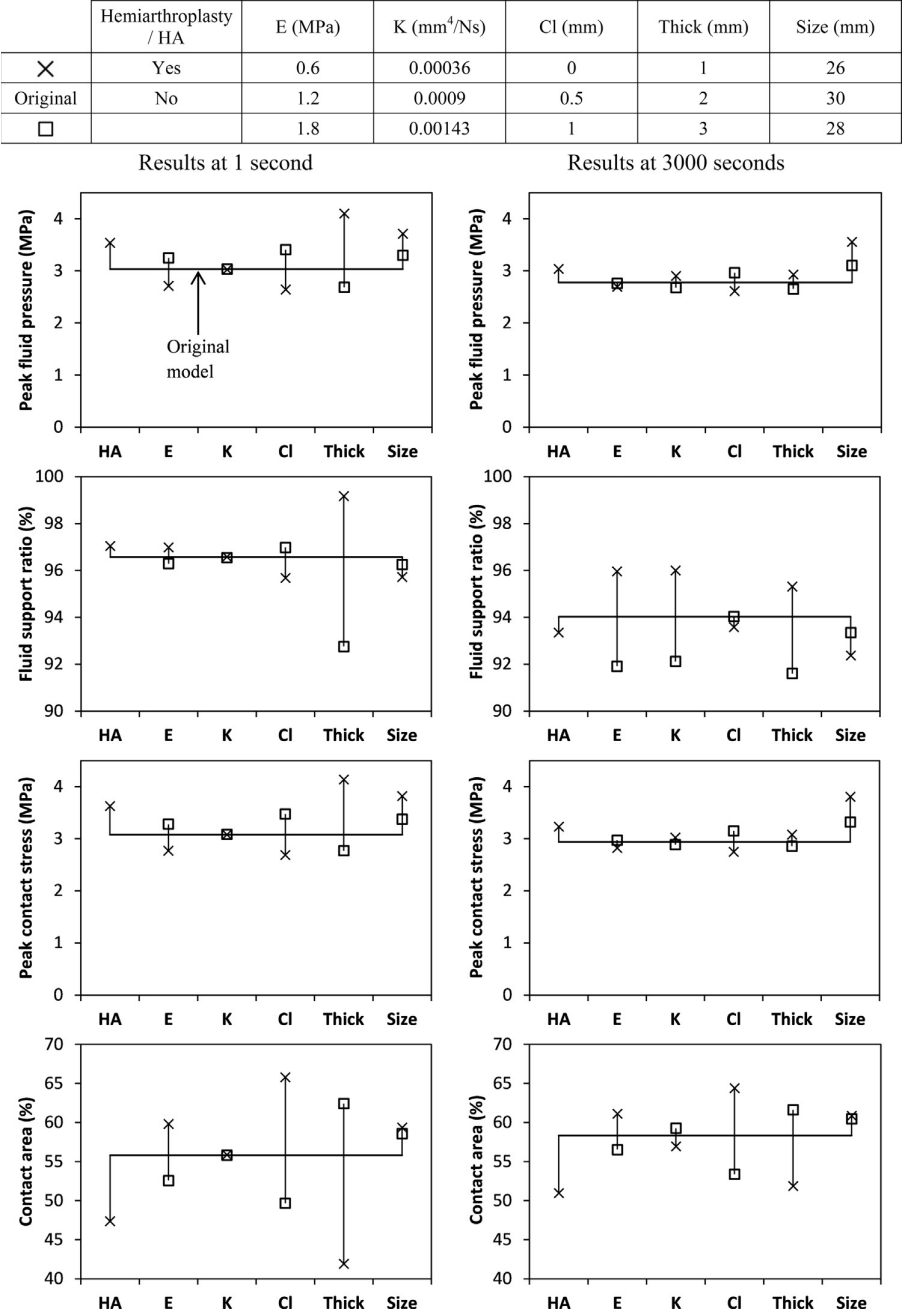
The results of the parametric tests for all models at 1 s and 3000 s. Both the short-term and long-term behaviour of the models depend on the size, clearance, hemiarthroplasty, cartilage thickness and stiffness. Cartilage permeability has almost no influence on the short-term behaviour, but greatly affects the long-term performance of the model.

**Fig. 5:**
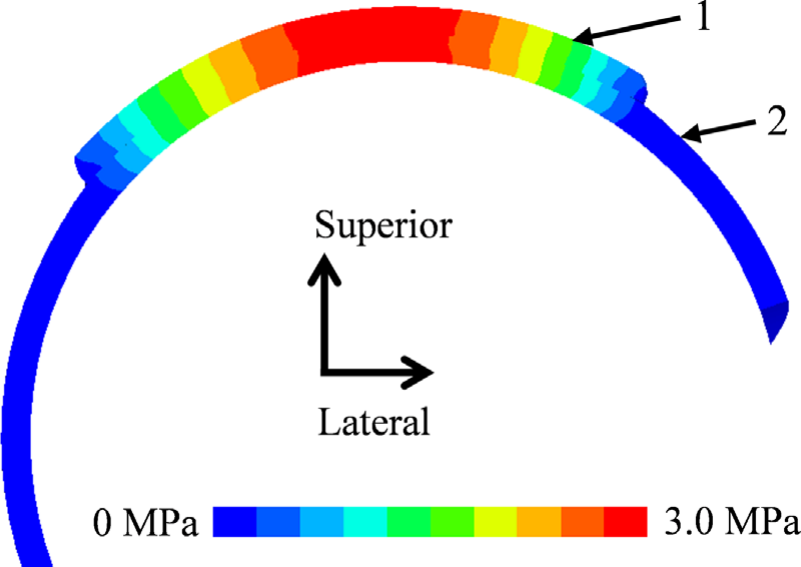
Cross-sectional view of fluid pressure (MPa) in the cartilage of the acetabulum (1) and femoral head and (2) of the original model at 1 s. Fluid pressure distribution was similar for the femoral head cartilage and acetabular cartilage. There was no marked difference in the fluid pressure across the thickness of the cartilage.

As an example, the fluid pressure distribution and contact stress of the original model are presented in Fig. 3. Over the acetabular cartilage surface, the contact stress and fluid pressure peaked around the centre of the cartilage and decreased gradually towards the edges. The contact stress and fluid pressure contours on both the femoral head and the acetabular cartilage surfaces of the natural hips were very similar. The peak fluid pressure was slightly lower than the peak contact stress over 3000 s for all the models (Fig. 4). There was no marked difference in the fluid pressure across the thickness of the cartilage (Fig. 5). The contact area was calculated as a ratio of the total surface area (3000 mm^2^ for the original model) of the acetabular cartilage available for articulation.

The results of the parametric studies are shown in Fig. 4. At the end of 1 s, the models with smaller size, larger clearance, stiffer cartilage aggregate, thinner cartilage or hemiarthroplasty had higher peak contact stress, higher peak fluid pressure and smaller absolute contact area. For all the models, the peak contact stress lay between 2.7 MPa and 4.1 MPa; the contact area ranged from 42% to 66%; and fluid supported 93% to 99% of the loads. At this early period after loading, the models with different cartilage permeabilities had nearly identical results.

Over the period of 3000 s, there was a decrease in the peak fluid pressure and the fluid support ratio for all the models. The models with larger size, stiffer cartilage aggregate, higher cartilage permeability, larger clearance, thinner cartilage or hemiarthroplasty had a greater decrease in the peak fluid pressure (Fig. 4). There was a decrease of over 10% in the peak fluid pressure for the models with 1.8 MPa cartilage Young's modulus, 0.00143 mm^4^/Ns cartilage permeability, 1 mm clearance and 1 mm thick cartilage, as well as the hemiarthroplasty case. Generally, the models with higher change in the peak fluid pressure also had higher change in the peak contact stress and contact area over 3000 s. For all the models, the reduction in the fluid support ratio was minimal and less than 5% even after 3000 s. As compared to the other parameters, changes in the fluid support ratio were most sensitive to the variation in cartilage permeability.

## 4 Discussion

In this study, a model of the whole natural hip with biphasic cartilage-on-cartilage and cartilage-on-solid contact was developed. Whilst such models have been employed previously for more simple geometry with two dimensions (Wu et al., 1997, Ferguson et al., 2000a, 2000b, Haemer et al., 2012), there are several challenges in representing the whole three dimensional joint and simulating the contact behaviour, particularly over a prolonged physiological period of loading. The approach taken in this study was to use FEBio to solve the models instead of Abaqus which has been used previously for the simulation of biphasic materials such as cartilage (Ferguson et al., 2000a, 2000b, Pawaskar et al., 2010, Manda et al., 2011). For contact problems with biphasic materials, the FEBio solver was able to achieve convergence in the simulation of the whole joint with biphasic cartilage-on-cartilage contact even over a prolonged period, which was not possible with other FE solvers. For example, Pawaskar (2010) employed Abaqus to simulate both the natural hip joint and the hip joint with hemiarthroplasty incorporating biphasic cartilage properties. Whilst the hemiarthroplasty model, which involved a rigid body on biphasic cartilage contact, could be simulated for 600 s under a static load, the natural joint model, also with spherical articulating surfaces, could only be solved for one second under a ramped load, due to convergence difficulties.

In this study, for the solid phase, a neo-Hookean model was adopted for practical reasons because the linearly elastic material within a biphasic model does not perform well with the non-linear FE solver in FEBio. This difference in material model is unlikely to affect the results because, across the range of strains seen in this study, it was found that the cartilage with a neo-Hookean solid phase in FEBio behaves nearly identically to the linearly elastic solid phase model in Abaqus in terms of the stress, strain and fluid pressure distribution (Fig. 2) (Maas and Weiss, 2007).

The primary aim of this study was to develop the necessary modelling methodology for simulating the natural hip over prolonged physiological periods. Whilst this was achieved, there were some limitations. In reality, as well as being biphasic, the cartilage layer is an inhomogenous fiber-reinforced structure (Mow et al., 1980, Soulhat et al., 1999, Ateshian et al., 2009), and the homogenous isotropic elastic model used here as a first approximation does not fully represent its behaviour. Although the 3000 s adopted in this study represents a relatively long physiological loading period, the cartilage behaviour is still relatively early in the transient phase and the results against time had not yet reached the equilibrium state that can be observed eventually in creep tests (Mow et al., 1980, Athanasiou et al., 1994). In terms of capturing the early stage response of creep tests, a tension–compression non-linear model may be more appropriate than the linear isotropic biphasic model which neglects the fact that the tensile modulus of the cartilage is substantially higher than its aggregate modulus (Soltz and Ateshian, 2000, Cohen et al., 1993, 1998). Consequently, the confinement effect due to the tensile stiffness may be reduced, and the peak fluid pressure, peak contact stress and fluid support ratio may be underestimated. The influence of cartilage thickness may also be amplified since here the confinement is provided more by the underlying bone geometry. Further development of this model will focus on the implementation of tension–compression non-linear solid phase into the whole joint model in order to evaluate these effects in more detail.

The congruence, size and material properties of the hip joint vary between individuals. The parametric study was therefore undertaken as a precursor to future model validation to identify the sensitivity of the model to these parameters. The findings of this study show that the contact mechanics of the hip joint are dependent on its congruence, size, cartilage thickness and properties as well as the contact type (i.e. cartilage-on-cartilage and cartilage-on-solid). Over the ranges studied here, the thickness and clearance were found to have the greatest effect on the contact mechanics. This is in agreement with the sensitivity study of Anderson et al. (2010) in an elastic model, where it was found that the cartilage thickness and local surface morphology had a major effect on the contact stress and distribution. Whilst the effect of the thickness may be overestimated by the simplified material model used, it is a parameter that needs to be taken into consideration in future sensitivity studies and subject-specific modelling.

The influence of the cartilage material properties was generally less than that of the morphology. In particular, the effect of the cartilage permeability on the contact mechanics of the hip joint was minimal during the early stages, but became evident after a period of load. The fluid support ratio was more sensitive to the cartilage thickness than other parameters at an early period because, as shown in Fig. 4, the hip congruence at this stage is highly related to the cartilage thickness as well as the clearance. For the model with thicker cartilage, the contact stress was spread more towards the area near the edge of the cartilage which is less confined than around the central region, leading to a lower fluid support ratio. This is because the fluid support ratio of the cartilage under unconfined compression is substantially lower than that under confined compression (Park et al., 2003, Ateshian and Hung, 2006). However, in reality, such differences may be reduced by the tension–compression nonlinearity of the cartilage. The hemiarthroplasty case showed higher peak stresses and a greater reduction in the fluid-load support over time than the cartilage-on-cartilage case. This illustrates that it is necessary to model both layers of cartilage to represent the natural joint since their interaction plays an important role in the contact mechanics.

For models with different parameters presented in this study, the predicted peak contact stress was found to range from 2.7 to 4.1 MPa. For similar loading conditions, the peak contact stress has been reported to lie between 4 MPa and 7 MPa in a study using embedded transducers (Brown and Shaw, 1983, Hodge et al., 1989) and between 5 MPa and 10 MPa in studies using pressure-sensitive films (Afoke et al., 1987, Anderson et al., 2008). Besides the linear isotropic assumption of the cartilage, the higher values of such measurements could be because the film thickness and stiffness introduce measurement artefacts, but also because of the smooth surfaces and regular morphology assumptions in this study, which have been shown to reduce the peak contact stress in an elastic FE model (Anderson et al., 2010). The peak stress predictions in this study are consistent with previous numerical studies where similar spherical assumptions have been made (i.e. 3 MPa to 4 MPa) (Mavčič et al., 2002, Yoshida et al., 2006, Pawaskar et al., 2010). For the purpose of the current study, the spherical assumption was necessary in order to undertake the initial parametric study and gain an understanding of the order of importance of the model input conditions. In order to directly validate the results against experiment using subject-specific models, it is clear that the individual variations in the morphology of the cartilage are important.

The labrum was excluded in this study due to a lack of extensive literature on its geometric parameters and material properties (Anderson et al., 2008), which is another potential limitation. Although the labrum plays a minimal role in load supporting for the normal hip (1–2% of total load) (Henak et al., 2011), it is believed to help impede the fluid exudation, owing to its lower permeability compared with the cartilage (Ferguson et al., 2000a, 2000b, 2003, Haemer et al., 2012). After labrum removal, the edge surface of the acetabular cartilage remains free-draining, potentially leading to a faster process of fluid exudation compared with a hip with the labrum. The findings in this study illustrate that even under the extreme situation where the labrum is removed, the fluid supports most of the load over prolonged physiological loading periods, further demonstrating the excellent function of the hip joint.

The primary advantage of the methodology in this study lies in its ability to investigate the solid phase and fluid phase separately, predict the joint tribological behaviour under both short-term and long-term loading periods, and interpret the influence of model parameters on the fluid-solid phases over prolonged physiological loading periods. Due to the importance of interstitial fluid in cartilage function and degeneration particularly over long-term loading periods (Mow et al., 1980, 1984, Ateshian et al., 1994), this modelling approach could allow further investigation of the functional and tribological behaviour of the joint and the pathology of joint degeneration. The results predicted by this study illustrate how the cartilage geometry and structure aid in the function of the natural hip joint. The soft and conforming contact surfaces ensure a large contact area and low peak contact stress, despite a high load being applied. Owing to the good congruence of the hip joint and the very low cartilage permeability, fluid exudation occurs slowly and the fluid supported most of the load even under extreme situations (e.g. 3000 s), leaving a small portion of load transferred to the solid phase of the cartilage and the solid-solid contact which would reduce the frictional coefficient and shear stress in reality (Krishnan et al., 2004).

In conclusion, in the present study a new method for simulating the contact mechanics and associated fluid pressurisation for a biphasic natural hip joint under prolonged physiological loading was presented. The predicted behaviour of the natural hip joint model was found to be subject to hip size, clearance, cartilage aggregate modulus, thickness and hemiarthroplasty for the period soon after loading. The fluid in the cartilage supports over 90% of the load transmitted between the articulating surfaces of the hip joint for a prolonged physiological loading period. The model with higher congruence or lower cartilage permeability has slower changes over this period. These findings are important for planning future subject-specific modelling approaches. Whilst there were some simplifications to the material model and geometry used in this study, the methods presented provide a basic platform and initial understanding of the sensitivity of the model onto which more sophisticated material models and geometric parameters could be added in the future. This computational approach has the potential to aid in understanding the mechanisms of hip function and the pathology of hip degeneration.

## Conflict of interest

None of the authors have any financial or personal relationships with other people or organisations that could have inappropriately influenced or biased the work.

## Figures and Tables

**Table 1:** The values of the parameters used in the original model and parametric tests. Only one parameter was altered from the original in each test case. E: Young's modulus of cartilage aggregate; K: cartilage permeability; Cl: radial clearance; Size: acetabulum radius; Thick: cartilage thickness.

**Model**	**E (MPa)**	**K (mm**^**4**^**/Ns)**	**Cl (mm)**	**Size (mm)**	**Thick (mm)**
**Original**	1.2	0.0009	0.5	30	2
**Values used in parametric studies**	0.6, 1.8	0.00036, 0.00143	0, 1	26, 28	1, 3
**References**	(Athanasiou et al., 1994)		(von Eisenhart et al., 1999)	(Xi et al., 2003)	(Shepherd and Seedhom, 1999)
